# Ultrasound-Guided Rotator Cuff Re-education for Residual Dysfunction After Shoulder Manipulation Under Ultrasound-Guided Cervical Nerve Root Block: A Case Report

**DOI:** 10.7759/cureus.92237

**Published:** 2025-09-13

**Authors:** Masashi Kawabata, Toru Miyata, Daichi Naoi, Kazuma Miyatake

**Affiliations:** 1 Department of Rehabilitation, Kitasato University School of Allied Health Sciences, Sagamihara, JPN; 2 Rehabilitation Center, Sagamihara Kyodo Hospital, Sagamihara, JPN; 3 Department of Orthopaedic Surgery, Yokohama City University, Yokohama, JPN

**Keywords:** frozen shoulder, manipulation under ultrasound-guided cervical nerve root block (muc), motor learning, rotator cuff, subscapularis, teres minor, ultrasound feedback

## Abstract

Shoulder manipulation under ultrasound-guided cervical nerve root block (MUC) can effectively restore the range of motion (ROM) in frozen shoulders; however, some patients experience persistent functional limitations. This case report describes a post-MUC rehabilitation program using ultrasound-guided real-time visual feedback to re-educate patients on rotator cuff contractions, complemented by targeted neural and muscle interventions. A recreational golfer in her 50s presented with frozen shoulder; ROM was limited to flexion 100°, abduction 80°, external rotation (ER) 20°, and internal rotation (IR) to L5, and she underwent MUC. At one month after the first MUC, the Disabilities of the Arm, Shoulder and Hand (DASH) decreased from 65 to 48 and ROM improved (flexion 165°, abduction 150°, ER 50°, IR to T12); at three months the DASH was 32, yet movement-evoked pain and motion apprehension persisted. A second MUC was performed; one month later the DASH was 15, but pain remained at 8/10 on the Numerical Rating Scale (NRS), dressing was infeasible, and golf practice/swing were not possible. She was referred to our clinic for physiotherapy. During the first session, ultrasound-guided contraction training immediately increased ER/IR strength from 2/10 to 7-8/10 on a patient-reported 0-10 scale, and movement-evoked pain decreased to 3-4/10 on the NRS. Ultrasound-guided manual therapy targeting the axillary nerve reduced apprehension in provocative positions and improved hand-behind-back motion. Subsequent training enhanced subscapularis strength in elevated position (bear-hug test: 3/10 → 7/10 on a patient-reported 0-10 scale). At 12 weeks, she returned fully to recreational golf, with a DASH score of 2 (sports module: 0). Ultrasound-guided real-time feedback with targeted neural and muscle interventions may facilitate neuromuscular re-education of the rotator cuff and bridge the gap between restored passive motion and functional performance after MUC.

## Introduction

Frozen shoulder, or adhesive capsulitis, is a condition in which the shoulder capsule thickens and contracts, causing progressive pain and stiffness with marked limitation of range of motion (ROM) [1,2]. Although conservative treatment for frozen shoulder is successful in up to 90% of patients [3,4], manipulation under anesthesia or manipulation under ultrasound-guided cervical nerve root block (MUC) is often indicated in refractory cases to restore mobility [5,6]. Although MUC can rapidly improve passive ROM, some patients continue to experience deficits in active function and sports performance [7].

MUC forcibly disrupts capsular adhesions to restore mobility [8]; however, this procedure may also cause iatrogenic injuries such as capsular tearing, labral injury, bone bruising, or even fracture [8-10]. Consequently, static stabilizers may be compromised, and dynamic stability becomes more dependent on the coordinated function of the rotator cuff. Therefore, persistent dysfunction after MUC is likely related to impaired rotator cuff control, highlighting the importance of targeted re-education in rehabilitation.

Residual dysfunction may be caused by impaired neuromuscular control of the rotator cuff [11]. Here, neuromuscular control refers to the timely and selective activation of the rotator cuff to maintain humeral-head centering and smooth shoulder motion. Neuromuscular exercises designed to enhance motor control have been shown in randomized controlled trials to produce superior improvements in pain and active ROM compared with strengthening exercises alone in patients with frozen shoulder [12]. However, no previous study has explicitly linked residual dysfunction after MUC to impaired neuromuscular control of the rotator cuff.

The teres minor and subscapularis are key stabilizers of the glenohumeral joint, and insufficient activation may limit their functional recovery [11]. Conventional rehabilitation protocols may not adequately address these selective activation deficits [1]. Real-time ultrasound imaging allows the visualization of muscle contraction and elongation during movement, provides immediate feedback to both patients and therapists, and may thereby enhance motor learning and targeted muscle re-education [13]. To the best of our knowledge, no previous study has described the use of ultrasound-guided visual feedback to retrain teres minor and subscapularis contractions after MUC. This case involves a recreational golfer with residual dysfunction after MUC, motivating an ultrasound-guided re-education approach to restore selective rotator-cuff activation and sports function.

## Case presentation

A female in her 50s, a recreational golfer, presented to another clinic with a frozen shoulder. On initial evaluation in the previous clinic, her active ROM was limited to 100° flexion, 80° abduction, 20° external rotation (ER), and internal rotation (IR) to the L5 level. The Disabilities of the Arm, Shoulder, and Hand (DASH) questionnaire was administered to evaluate upper extremity function (baseline total: 65; sports module: 100). The DASH questionnaire is a 30-item patient-reported outcome scored 0-100 (higher scores indicate greater disability). The four-item sports module is also reported (0-100), and a change of 10-15 points is considered the minimal clinically important difference (MCID) [14]. The DASH was originally developed by Hudak et al. [15], and the validated Japanese version was reported by Imaeda et al. [16]. The patient then underwent MUC.

One month after the first MUC, her ROM improved to 165° flexion, 150° abduction, 50° ER, and IR to T12 (Table 1). The DASH total decreased to 48, but the sports module remained 100. At three months after the first MUC, the DASH total further decreased to 32, exceeding the MCID; however, compared with the prospective series [6] (mean DASH 31.8±15.4 pre-MUC to 16.2±10.6 at three months and 10.2±12.9 at one year after MUC), her recovery appeared slower. Because movement-evoked pain and fear during essential activities of daily living (ADLs) - particularly dressing - persisted, a second MUC was performed. One month later, the DASH total had improved to 15, yet movement-evoked pain persisted (Numerical Rating Scale [NRS] 0-10: 8), the sports-module score remained 100, and the patient was far from sports readiness. She was therefore referred to our clinic to initiate physiotherapy aimed at functional restoration.

**Table 1:** Clinical course and outcomes Pain intensity (movement-evoked)—Numerical Rating Scale (NRS, 0–10; 0 = no pain with movement, 10 = worst pain with movement). Relative strength—patient-reported 0–10 scale anchored to the contralateral limb (=10) PT, physiotherapy; MUC, manipulation under ultrasound-guided cervical nerve root block; ER, external rotation; IR, internal rotation; US-FB, ultrasound-guided visual-feedback; SSc, subscapularis; ADLs, activities of daily living; Ext, Extension; Abd, abduction; NRS, Numerical Rating Scale; ROM, range of motion; L, lumbar vertebral level; T, thoracic vertebral level; DASH, Disabilities of the Arm, Shoulder and Hand

Time point		Previous clinic	→	→	→	→	Our clinic	PT 1st session	PT 3rd session	PT 4th session	PT 8th session	PT 9th session	End session
Parameter		Pre MUC		1month after 1st MUC	3months after 1st MUC		1month after 2nd MUC	(immediaterly effect)	(week2)	(week3)	(week7)	(week8)	(week12)
Key Intervention			1st MUC			2nd MUC		ER/IR with US-FB exercise.	axillary-nerve–focused manual therapy			SSc with US-FB exercise	
Functional changes							Pain and fear with unavoidable ADLs such as dressing	Marked reduction in fear of movement and improved ER/IR strength.	Ext–Abd–IR without apprehension	Daily-life fear resolved	Fear of golf swinging	Partial return to golf	Full return to golf
Pain (NRS 0-10)							8	3–4		0–1			
ROM (°)	Flexion	100		165								170	
	Abduction	80		150								170	
	ER	20		50								60	
	IR	L5		T12								T10	
Relative Strength (0–10)							ER/IR 2	ER/IR 7–8				Elevated IR (3 to 7–8)	
DASH score (0-100)		65		48	32		15		20	16	16	15	2
DASH sports module (0-100)		100		100	100		100		100	100	100	56	0

Ultrasonographic muscle dynamic assessment (pre-treatment)

**Figure 1:**
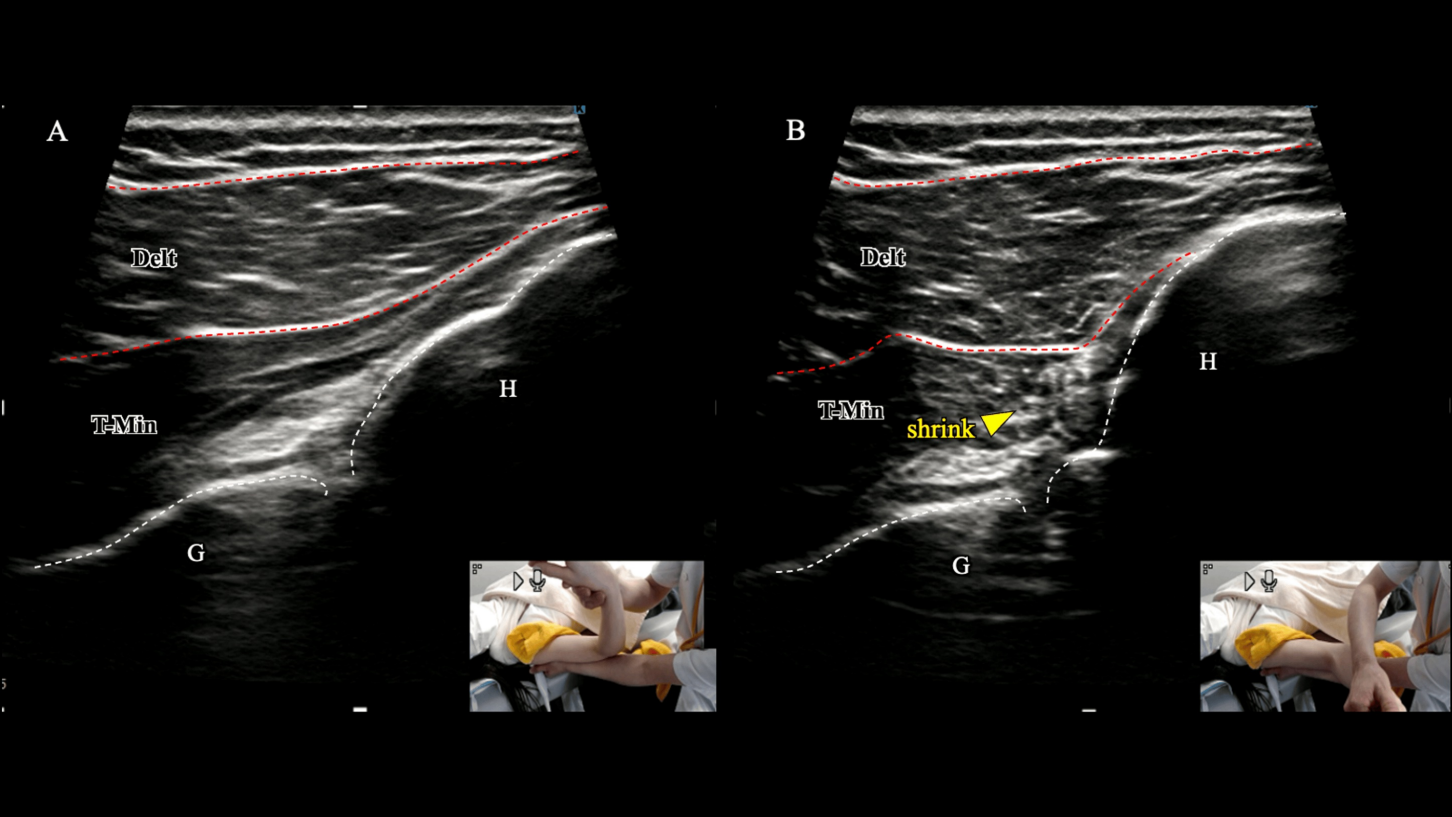
Ultrasonographic assessment of teres minor dynamics during external rotation (ER). During active-assisted ER from the start (A) to end range (B), the teres minor (T-Min) shows localized tissue folding/buckling with reduced excursion along the posterior glenohumeral joint, coinciding with the patient’s stiffness and perceived weakness. T-Min, teres minor; Delt, deltoid; G, glenoid; H, humeral head

Dynamic ultrasound imaging was used to evaluate the teres minor and subscapularis muscles during active assistive movements in the first session. All ultrasonography was performed by a licensed physiotherapist (17 years clinical experience; four years musculoskeletal ultrasound experience). Under normal contraction, sonography demonstrates uniform thickening/shortening with increased pennation and smooth fascial-plane gliding. By contrast, abnormal responses include localized tissue buckling/folding, reduced excursion, and compensatory activation of adjacent muscles-features we used to identify selective activation deficits. During ER, the teres minor displayed localized tissue folding and reduced excursion in the posterior aspect of the glenohumeral joint, which coincided with the patient’s sensation of stiffness and perceived weakness (Figure 1 and Video 1).

**Video 1:** Ultrasonographic assessment of teres minor dynamics during external rotation (corresponds to Figure 1). Real-time ultrasonographic assessment of teres minor during active-assisted external rotation, illustrating posterior tissue folding/buckling and reduced excursion at the glenohumeral joint.

**Figure 2:**
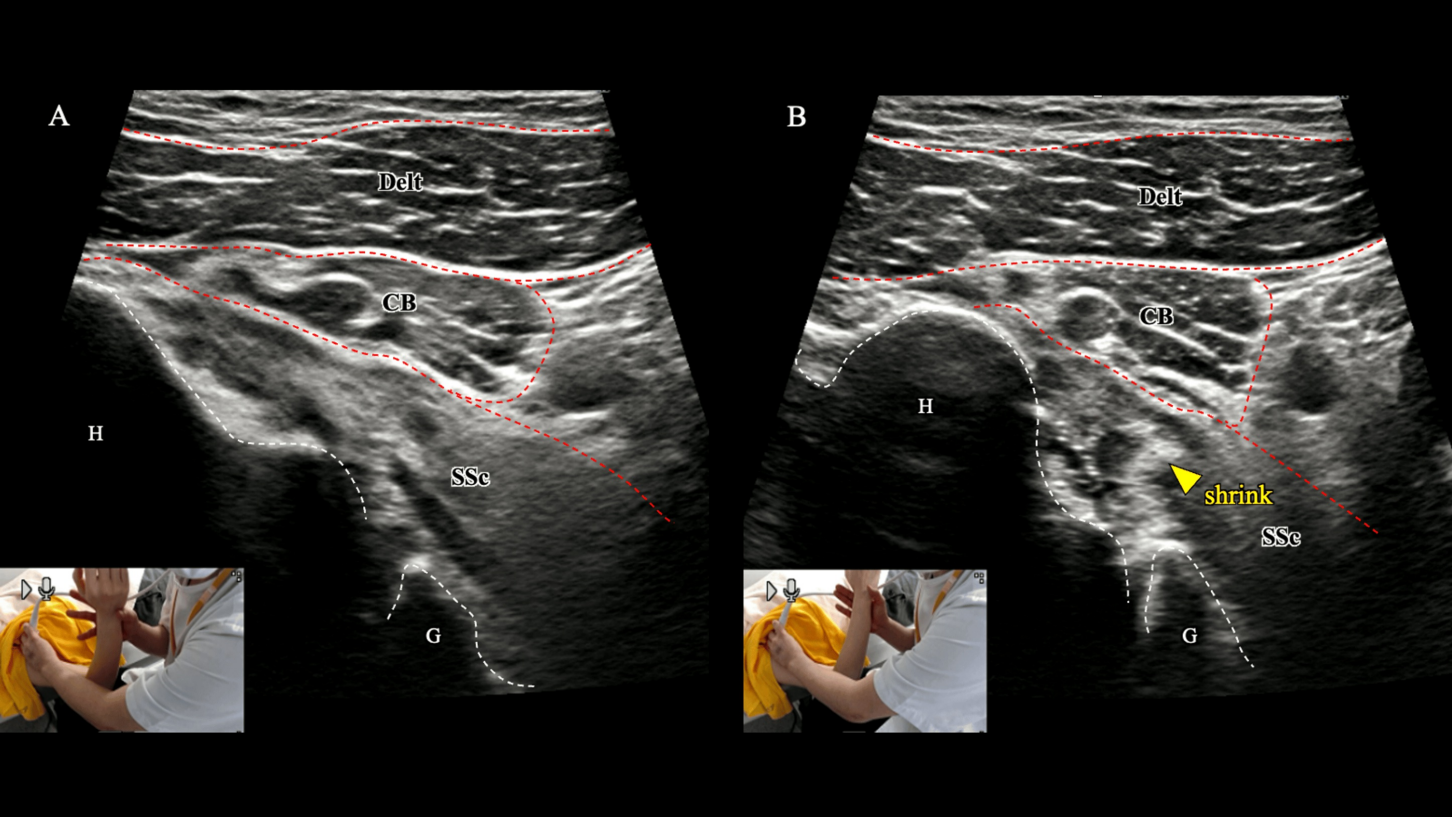
Ultrasonographic assessment of subscapularis (SSc) dynamics during internal rotation (IR). During active-assisted IR from (A) to (B), the SSc shows anterior tissue folding, incomplete humeral-head translation at end range, and compensatory activation of other internal rotators, consistent with the patient’s perceived weakness. Delt, deltoid; CB, coracobrachialis; G, glenoid; H, humeral head.

During IR, the subscapularis similarly demonstrated tissue folding on the anterior aspect, with incomplete humeral head translation to the end range and visible compensatory activation of other internal rotators (Figure 2 and Video 2). These sonographic findings indicate impaired selective recruitment and compromised dynamic stability and directly informed the subsequent physiotherapy plan. In settings without ultrasound, comparable deficits may present clinically as an impingement-like blocking or pain at end range, and as seemingly preserved strength in the mid-range with disproportionate weakness in the shortened position, often accompanied by compensatory scapulothoracic or deltoid overactivity. Recognizing this pattern can still guide targeted neuromuscular re-education even when imaging is unavailable.

**Video 2:** Ultrasonographic assessment of subscapularis dynamics during internal rotation (corresponds to Figure 2). Real-time ultrasonographic assessment of subscapularis during active-assisted internal rotation, showing anterior tissue folding, incomplete humeral-head translation at end range, and compensatory activation of other internal rotators.

Physiotherapy intervention

**Figure 3:**
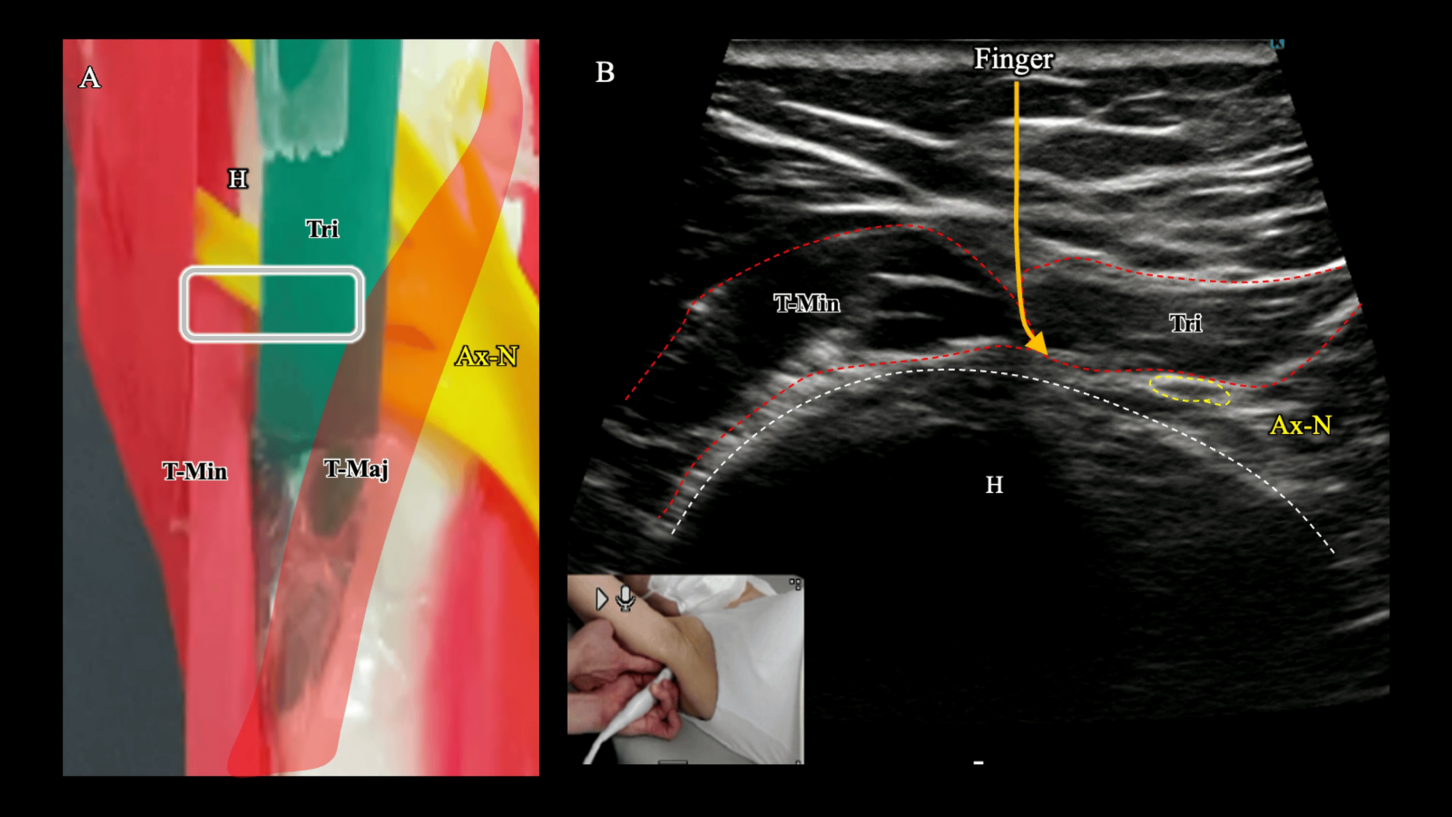
Ultrasonographic image and schematic of the quadrilateral space (QLS). (A) Author-created schematic based on a shoulder model. (B) Corresponding ultrasound image. Palpation-based soft-tissue mobilization was performed in the QLS, with the therapist placing a finger gently between the teres minor (T-Min) and the triceps brachii (Tri) to mobilize interfascial tissues and orient toward the axillary nerve (Ax-N) located between the Tri and the humeral head (H). The arrow indicates finger placement. T-Min, teres minor; T-Maj, teres major; Tri, triceps brachii; Ax-N, axillary nerve; H, humeral head

The rehabilitation program was tailored session-by-session, with interventions selected based on the patient’s primary complaints and functional findings observed through real-time ultrasound evaluation. The key ultrasound-guided interventions and their corresponding outcomes are summarized in Table 1. The program began with ultrasound-guided real-time visual feedback contraction training for the teres minor and subscapularis muscles during active-assisted and end-range ER and IR exercises for five to seven minutes, conducted immediately following the assessment in the first session. In the third session, palpation-based soft-tissue mobilization was performed in the quadrilateral space (QLS) region, with the therapist placing a finger gently between the teres minor and the long head of the triceps to mobilize the interfascial tissues (Figure 3). For safety, care was taken to avoid compression of the posterior circumflex humeral artery and to continuously monitor pain and neurovascular status throughout the procedure. In the ninth session, ultrasound-guided contraction training of the subscapularis in an elevated IR position was implemented.

Outcomes

**Figure 4:**
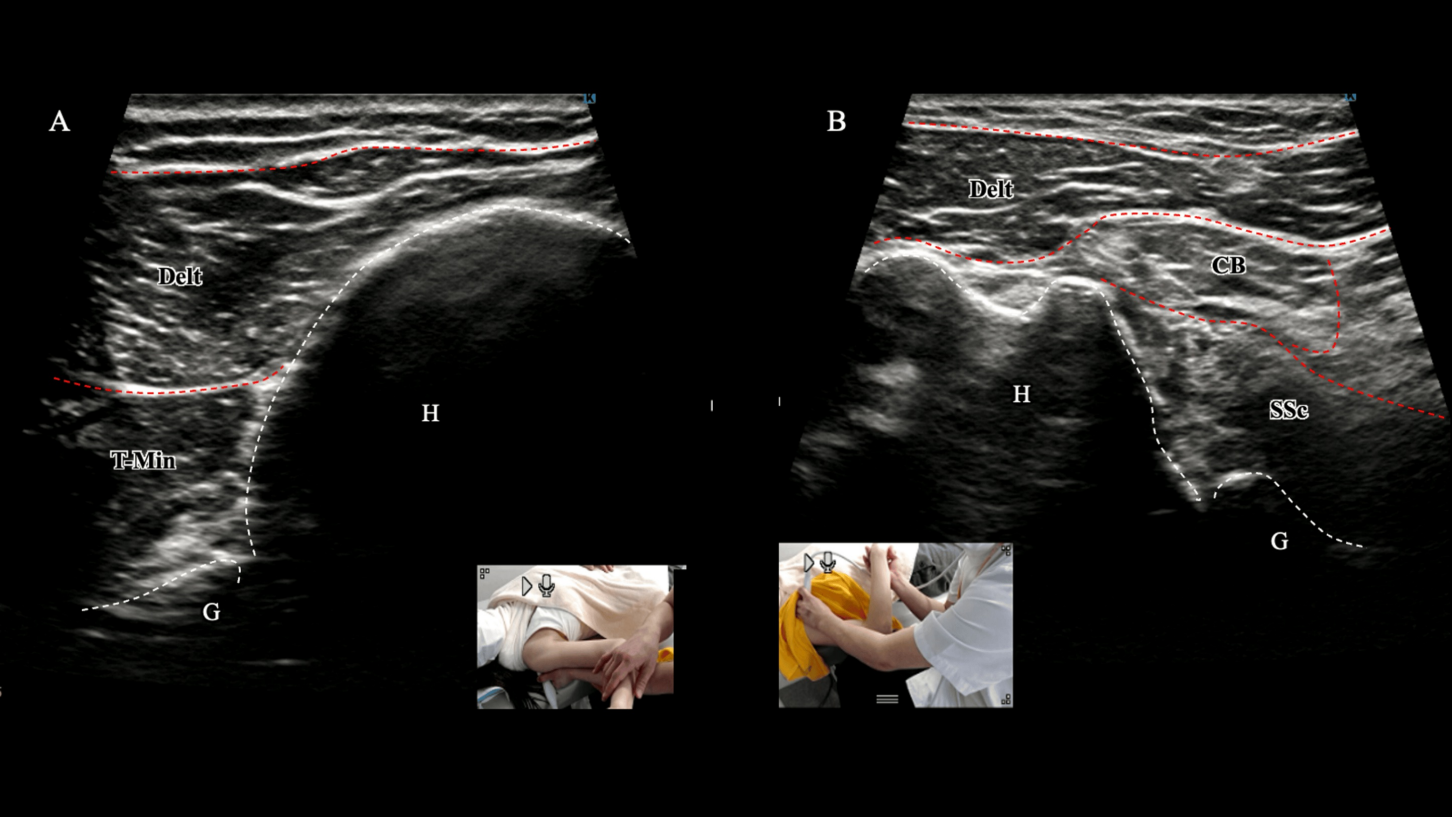
Post-training dynamics of the teres minor (A) and subscapularis (B). After repeated ultrasound-guided visual-feedback contraction exercises, the T-Min during external rotation (ER) shows smooth thickening/shortening without localized folding, and the SSc demonstrates greater end-range internal rotation (IR) excursion with improved awareness/selective activation. These changes paralleled the clinical improvements in ER/IR strength and movement apprehension. Delt, deltoid; T-Min, teres minor; SSc, subscapularis; CB, coracobrachialis; G, glenoid; H, humeral head

During the first rehabilitation session, patient-reported strength (0-10), anchored to the contralateral limb (=10) and collected for ER/IR, improved immediately from approximately 2 to 7-8 following ultrasound-guided contraction training (Figure 4 and Videos 3, 4). In the third session, after axillary-nerve-focused manual therapy via the quadrilateral space (see Figure 3), apprehension during ER, abduction, and IR decreased immediately and the effect persisted through the fourth session; fear during daily activities, including dressing, had resolved. Movement-evoked pain and fear during ADLs decreased to NRS 0-1 immediately after the third session and remained at this level at the fourth session; the patient explicitly reported no difficulty with dressing. At that point, however, she continued to avoid golf practice and reported fear of swinging, so a graded return-to-golf program was established as the next goal. By the ninth session, after continued subscapularis training in the elevated position, bear-hug test strength (palm on the contralateral shoulder with the elbow anterior to the trunk; resisting the examiner’s attempt to lift the hand off the shoulder) improved from 3 to 7-8 on the patient-reported 0-10 scale. The DASH total showed a clinically meaningful improvement exceeding the MCID; the sports-module score improved progressively and, by week 12, coincided with a full return to golf.

**Video 3:** Dynamics of the teres minor during external rotation after repeated ultrasonographic feedback contraction exercises (corresponds to Figure 4A). Post-training teres minor during external rotation after ultrasound-guided visual-feedback exercises, demonstrating uniform thickening/shortening without localized folding.

**Video 4:** Dynamics of subscapularis during internal rotation after repeated ultrasonographic feedback contraction exercises (corresponds to Figure 4B). Post-training subscapularis during internal rotation (IR) after ultrasound-guided visual-feedback exercises, demonstrating increased end-range excursion and improved selective activation.

## Discussion

MUC is effective in improving the ROM in a frozen shoulder; however, residual deficits in strength, motor control, and movement confidence are common even after passive mobility is restored [7,8]. This patient represented such a scenario: the patient achieved near-full ROM after MUC, yet continued to experience pain during activities and movement apprehension. Relative to prospective trajectories after shoulder manipulation [6], in which DASH scores typically approach the mid-teens by three months, this patient’s functional recovery lagged despite MCID-exceeding improvement. This “ROM recovered but function lagging” gap motivated a targeted, ultrasound-guided rehabilitation approach.

Ultrasound-guided contraction training provided immediate visual feedback on muscle activation. This allowed both the patient and therapist to directly observe whether the rotator cuff contraction continued smoothly to the end ROM or whether an error had occurred. Such feedback is a critical element in performing complex motor tasks because it enables error detection, correction, and reinforcement of appropriate movement strategies [13,17].

In this patient, both ER and IR strengths improved dramatically during the first session. This likely reflects the re-establishment of neuromuscular control in the teres minor and subscapularis muscles. These muscles act reciprocally to stabilize the humeral head in the glenoid fossa during rotation [18]. Cadaveric biomechanical studies have demonstrated that the teres minor and subscapularis resist superior humeral head migration, thereby preserving glenohumeral stability [18]. Their activity is tightly coordinated and innervated by branches of the posterior cord-the axillary nerve for the teres minor and the upper and lower subscapular nerves for the subscapularis [19]. Thus, dysfunction of either muscle disrupts balance, leading to humeral head translation, compensatory strategies, and loss of functional strength. Targeted re-education in this rehabilitation program likely restored not only contractile strength but also the physiological coordination of these muscles, thereby contributing to functional stability. Clinically, such training can help close the residual gap between restored passive mobility and sports performance.

In addition to muscular dysfunction, impaired axillary nerve mobility may contribute to persistent deficits following MUC. The axillary nerve traverses the QLS between the teres minor and triceps muscles and wraps around the humerus from the posterior to the anterior side [19]. During extension, abduction, and IR, the position required for the hand-behind-back motion (the nerve) is stretched. If entrapment or adhesions occur in the QLS, gliding of the axillary nerve may be restricted, leading to excessive tension, pain, and fear of movement. In this patient, ultrasound-guided manual therapy targeting the axillary nerve reduced apprehension during the hand-behind-back motion, suggesting that neural mobilization relieved tension and facilitated improved recruitment of the teres minor and subscapularis muscles, thereby enhancing functional confidence.

From a behavioral perspective, early and visible improvements appeared to increase the patient’s self-efficacy, which likely promoted adherence and engagement throughout the rehabilitation process. Such responses are consistent with motor learning theory, which emphasizes the importance of immediate reinforced feedback in acquiring and retaining complex motor skills [17].

This is a single-patient report with short follow-up, limiting generalizability. Substantial gains in ROM and total DASH had already occurred before the ultrasound-guided program, and a second MUC was performed; therefore, the incremental effect of our rehabilitation cannot be isolated from natural recovery or standard care. Baseline radiographs and MRI were unavailable because the manipulation was performed at another institution; functional sonography was used instead to characterize residual impairment after MUC. Strength outcomes were patient-reported and likely reflect neuromuscular re-education rather than hypertrophy; handheld dynamometer measurements were not collected. Objective assessments of muscle activation and movement were not obtained. Future studies should recruit larger cohorts, extend follow-up, include quantitative measures of muscle activation and nerve mobility, and use comparative designs to isolate the specific contributions of ultrasound-guided contraction training and neural mobilization after MUC.

## Conclusions

Ultrasound-guided rotator cuff re-education appeared effective in resolving residual dysfunction after shoulder MUC in this patient and may facilitate neuromuscular re-education. This case highlights the importance of targeted neuromuscular training in restoring functional stability and enabling return to sports. Further studies with larger cohorts are warranted to confirm the generalizability and long-term efficacy of this approach.
